# Response of ceramic microbial fuel cells to direct anodic airflow and novel hydrogel cathodes

**DOI:** 10.1016/j.ijhydene.2019.04.024

**Published:** 2019-06-07

**Authors:** J. Winfield, J. Greenman, I. Ieropoulos

**Affiliations:** Bristol BioEnergy Centre, Bristol Robotics Laboratory, University of the West of England, Bristol, UK

**Keywords:** Microbial fuel cell, Wastewater treatment, Hydrogel, Biosensor, Bioelectrochemical system, Sequencing batch reactor

## Abstract

The presence of air in the anode chamber of microbial fuel cells (MFCs) might be unavoidable in some applications. This study purposely exposed the anodic biofilm to air for sustained cycles using ceramic cylindrical MFCs. A method for improving oxygen uptake at the cathode by utilising hydrogel was also trialled. MFCs only dropped by 2 mV in response to the influx of air. At higher air-flow rates (up to 1.1 L/h) after 43–45 h, power did eventually decrease because chemical oxygen demand (COD) was being consumed (up to 96% reduction), but recovered immediately with fresh feedstock, highlighting no permanent damage to the biofilm. Two months after the application of hydrogel to the cathode chamber, MFC power increased 182%, due to better contact between cathode and ceramic surface. The results suggest a novel way of improving MFC performance using hydrogels, and demonstrates the robustness of the electro-active biofilm both during and following exposure to air.

## Highlights

• Ceramic cylindrical microbial fuel cells subjected to airflow through anode chamber.
• Impact minimal with drops of <2 mV during 4 h spells of air flow.
• Power did decline at higher air-flow rates because COD reduction accelerated.
• No ill-effect after air exposure; recovery was immediate when fed fresh feedstock.
• Expanding hydrogel with catholyte improved electrical output by 182%.

## Introduction

The generation and subsequent treatment of wastewater will always be a challenge to humanity. Human health and the state of the environment are under threat from pollution of the waterways and for this reason there have been significant efforts to improve treatment technologies in terms of both efficiency and cost reduction. Microbes are utilised in a number of technologies including aerobic treatment e.g. biological filters and anaerobic such as anaerobic digestion. Another technology that can complement these is the microbial fuel cell (MFC) and research efforts have grown over the past couple of decades such that there are now a number of realistic target applications. The technology can offer electricity and sanitation in remote locations as demonstrated through successful field trials using urine as fuel [30]. MFCs can be configured into systems that provide a sufficient and constant supply of energy to power useful devices such as robots [23], meteorological buoys [42], pumps [20], mobile phones [13] and transceivers [32]. While these are exciting advances, perhaps the area that is most suited to the scale-up of MFCs is wastewater treatment. MFCs have demonstrated in numerous studies over the last decade that wastewater with diverse and complex compositions can be utilised as fuel [12]. The MFCs adapt and can deal with different types of waste that vary considerably in parameters such as organic loading [18], pH [35], conductivity [43], sulphide content [27], toxicity [26] and other factors. Given that the fuel can be any organic waste liquid, which is treated whilst electricity is generated, there is a real focus on scaling up the technology for wastewater treatment.

Where MFCs might fit into the treatment process is still cause for debate. To date they have functioned efficiently when fed industrial strength wastewater with high organic loading i.e. of the type that might be found at the beginning of the treatment process [40]. MFCs have also shown to be suitable for operating at the end of a multi-stage process where the effluent is weaker and with low chemical oxygen demand (COD), demonstrating that they could perhaps function as a polishing stage [44]. In addition to the composition of the wastewater itself, other factors can affect MFC behaviour, potentially to the detriment of performance. For example, the flow of water will introduce oxygen and in addition, processes such as the sequential batch reactor (SBR) actively introduce air. Therefore if a MFC was to function in such an environment, it needs to demonstrate resilience to the influx of air. The SBR process is multi-stage and involves anoxic and aerobic stages and MFCs can complement the process by improving treatment efficiency when placed downstream [38], demonstrating in this way the treatment of pharmaceutical wastewater [39]. However, while it is accepted that MFCs can function in an anoxic environment, it is not clear how they behave when exposed to air, fed directly into the anode chamber. The current study used cylindrical ceramic MFCs in order to study their behaviour under active aeration conditions.

### Oxygen in the anode and anolyte flow rate

Ceramic MFCs are a cost effective option for scale up [34] and this study looked at their behaviour in a fluctuating environment when air was purposely pumped through the anode chamber alongside the flow of anolyte.

The presence of oxygen in the anode chamber could be a problem because dissolved oxygen is found in the treatment process particularly at high flow rates [16]. Key for MFC operation is the transfer of electrons from within the bacterial cell to the anode surface. When oxygen is present in the anode chamber, microorganisms that are metabolically compatible (i.e. aerobic and facultative anaerobic) will oxidise the fuel with the reduction of oxygen rather than using the electrode [25]. While this does not affect the treatment efficiency, it will affect the overall rate of microbial metabolism, power generation and coulombic efficiency. In addition, the presence of oxygen is toxic to strictly anaerobic microorganisms, which in the case of MFCs, could also result in a sub-optimal electrical output. A small number of studies have looked at the influence of oxygen on MFC behaviour [28] and showed that its did not significantly affect power using electro-active monocultures such as the facultative organism *Shewanella oneidensis*. For this species the presence of oxygen limited the rate of electron transfer to the anode but it also promoted biofilm development, which counteracted the negative impact [22]. *Geobacter sulfurreducens* is another well-studied electro-active organism and was long thought to be a strict anaerobe, yet it can in fact grow using oxygen as terminal electron acceptor [21] and can produce power in a MFC when oxygen is present providing it is fed from within the anode [25]. A long-term study was performed by De Sá et al [5] who directly exposed areas of the surface of the anode to air. They found a linear relationship between the amount of surface exposed and the degree of inhibition observed by the biofilm.

For operation *in situ* within a wastewater treatment plant, the anodic biofilm will always be a mixed community. Even assuming that the MFC system begins with a single species, over time local organisms in the feedstock are thought to co-colonise or accumulate within the microbial biofilm community, with the whole electrode evolving to suit the prevailing physicochemical conditions. Furthermore, because wastewater is a complex substrate, only a mixed microbial community operating synergistically could deal with the wide mix of organics. Therefore, it is important to know how oxygen might affect such a mixed community. Oh et el. (2009) looked at the effect of oxygen penetrating the proton exchange membrane (PEM) using H-type MFCs being batch-fed [45]. They demonstrated that oxygen did cause the power to drop but that MFCs quickly recovered when the airflow stopped. PEMs can inhibit electro-active bacteria in some conditions [33] and the current study focussed on mixed communities in ceramic MFCs where conditions for electron-abstraction by the anode are more favourable.

Moving MFC materials and design away from the conventional H-type and/or cubic type has seen the technology advance from laboratory curiosities towards successful field trials [15]. One of the main factors has been the adoption of ceramic as both the structural material and as medium for ion transfer between the anode and cathode. Initial work looked at pots and cylinders that were configured with the anode inside the vessel and the cathode wrapped around the outside [1], [2], [36]. More recently, a commonly used design incorporates the cathode inside the cylinder (sealed at the bottom) and the anode wrapped around the outside [8]. MFCs of this configuration allow the cylinder to be immersed in the anolyte reservoir with the anode exposed to the liquid whilst the cathode sits open to the air. The current study used this design of ceramic cylindrical MFCs and mixed biofilms to explore how they respond to the influx of air directly into the anode chamber.

In addition to the introduction of air, the flow rate and hydraulic retention of anolyte is an important consideration. Recently an electrochemical technique for analysing the suitability of materials and operations has been the bi-directional polarisation sweep and this method was incorporated into the study in order to analyse the effects of the flow rate of anolyte on long-term power output performance by the MFCs.

### Optimising the air-breathing cathode in cylindrical ceramic MFCs

The internal cathode design allows liquid catholyte to accumulate, which improves the performance by lowering the ohmic resistance and preventing biofouling [10]. An unresolved engineering challenge with this design has been the poor contact of the cathode with the inner wall of the cylinder. The damp environment can cause the electrode to peel away or become loose as reflected in a drop in power. Another challenge is affixing and selecting the best material for connecting the current collector. Different clays vary in their chemical make-up and so one current-collecting wire might be suitable for one ceramic type but not another. This is because components in the ceramic might be more prone to reacting with the wire materials. In an attempt to address these issues, a unique method was trialled to improve cathode contact by introducing hydrogel powder into the cathode chamber.

## Methods and materials

### MFC construction and operation

Large terracotta MFCs (120 mL volume) in triplicate were employed for all experiments. For the airflow experiments the large MFCs were compared to a smaller size ceramic MFC (40 mL volume) (in triplicate) to demonstrate the role of chamber volume. The dimensions and configurations of each MFC type are detailed in Table 1.

**Table 1:** Size and dimensions of the two MFCs types.

MFC	Outer surface area (cm^2^)	Inner surface area (cm^2^)	Cathode chamber volume (mL)	Anode chamber volume (mL)
Large	166	138	103	120
Small	42	33	13	40

All ceramic cylinders were sealed at one end (the base). Cathodes were prepared as previously described [11] by coating a carbon veil sheet with 30% PTFE (Sigma Aldrich). When dry, the material was spatula-coated with an activated carbon and PTFE mixture, which was prepared by mixing 80g of activated carbon powder (G. Baldwin and Co, UK) with 20 wt% PTFE (60% PTFE dispersion in dionised water). The AC/PTFE mixture and carbon veil were hot pressed at 150–200 °C using a household iron. When dry, the cathodes were cut into sizes so that they completely covered the internal cylinder walls with the activated carbon layer facing the ceramic and the PTFE layer facing outwards open to air. Piano wire wrapped in stainless steel was bent inside the chamber to push the cathode against the wall and enable connection to the data logging equipment.

**Fig. 1:**
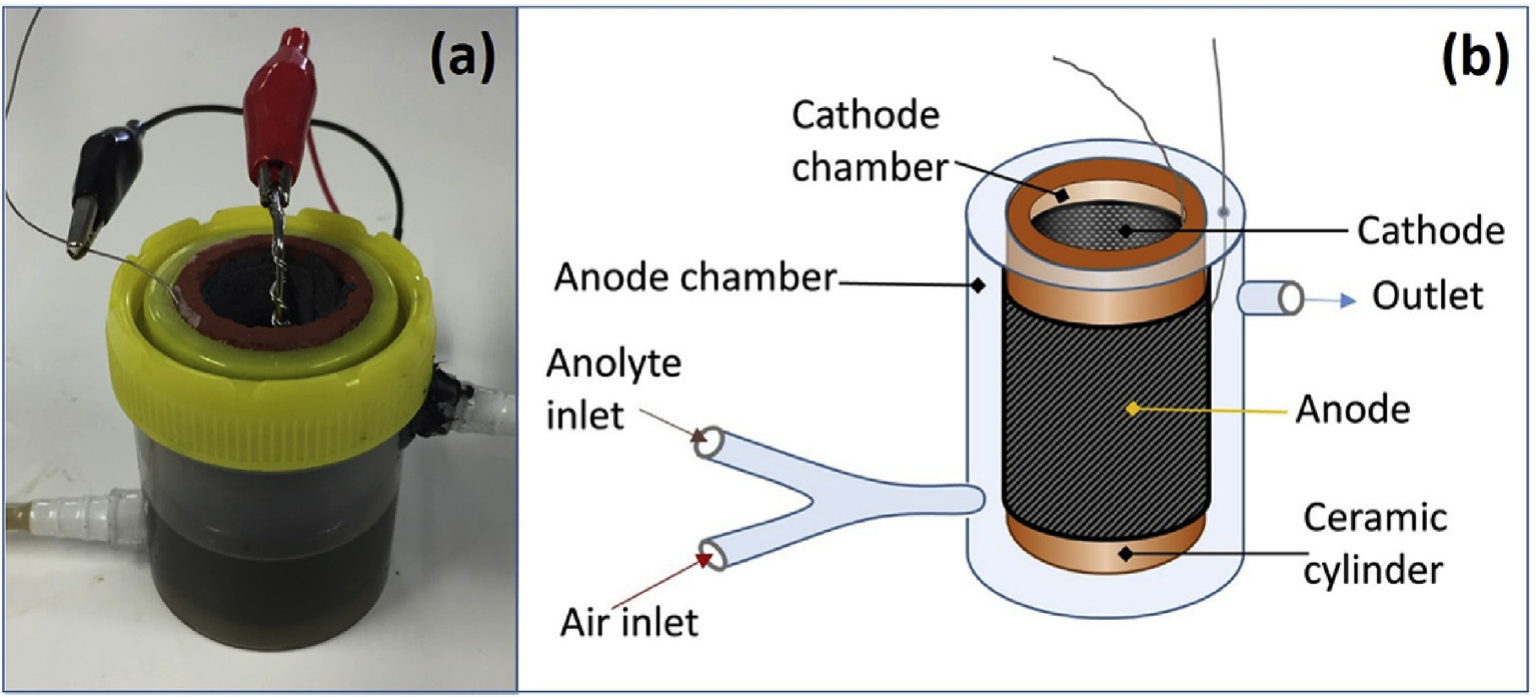
(a) Photograph and (b) schematic of the ceramic MFC used in study.

The anodes were pieces of carbon veil wrapped around the outside of the ceramic cylinders. For the large MFC a 20 × 60 cm (1200 cm^2^) piece of carbon veil was used and for the smaller MFCs 10 × 40 cm 400 cm^2^. To ensure the carbon veil was secured firmly to the surface, it was wrapped at the top and bottom with a strip of parafilm. To act as current collector for the attachment of crocodile clips, stainless steel wire was threaded through the carbon veil. The MFCs were sealed into plastic containers using an aquarium sealant (Wet Water Sticky Stuff, Barry Read Supplies, UK) (see Fig. 1a). Inlet (bottom) and outlet tubes (top) were incorporated into the plastic anode chambers so that the MFCs could be operated in continuous flow. Two routes into each MFC were available through incorporation of a Y-junction at the inlet (Fig. 1b). One inlet was for anolyte and the other for the pumping of air into the chamber. Two multi-channel peristaltic pumps (Watson Marlow 205U) were used; one to channel in air and the other anolyte.

MFCs were inoculated using activated sludge (Wessex Water, Cam Valley, Bristol) enriched with tryptone (1%) and yeast extract (0.5%). Following inoculation and for all experiments synthetic wastewater was used, consisting of 20 mM sodium acetate and 0.05% tryptone and yeast extract (TYE) with a COD of approximately 1720 mg O_2_/L. Unless otherwise stated for all experiments MFCs were run in closed circuit conditions under a 500 Ω fixed external resistance.

#### Airflow into anode chamber

To investigate the impact that atmospheric oxygen had on the health of the electroactive biofilm, air was fed into the anodic chamber. Air was used as opposed to pure oxygen because it better mimicked the aerobic wastewater treatment processes and therefore better illustrated how MFCs might behave in such an environment. Air was introduced into the anode chamber either in a one off injection with a syringe (See section: Single injection of air directly into anode chamber) or via a peristaltic pump (See section: Four hours on/four hours off flow of air into anode chambers). Air was pumped into the anode chamber for 4 h before anolyte was pumped for the next 4 h then the cycle started again. Timer switches controlled the pumps to ensure a continuous cycle. Anolyte was always fed at a flow rate of 5.2 mL/h. Three different air-flow rates were investigated; 126 mL/h, 252 mL/h and 1.1 L/h. In addition, an experiment was carried out to look at the effect of pumping air into the anolyte reservoir at 126 ml/h (See section: Air pumped into the reservoir bottle).

#### Anolyte flow

In order to look at the bio electrochemical effect of two different hydraulic retention times (HRTs) on the biofilm, the larger 120  mL MFCs were employed because their size in combination with the flow rates, permitted the desired range of HRTs. During the anolyte flow rate experiment (See section: Effect of anolyte flow rate on bi-directional polarisation), no air was pumped but the anolyte was fed through the MFC chambers at either 30 mL/h (HRT of 4 h) or 5.2 mL/h (HRT of 23 h). Bi-directional polarisation sweeps were run at these flow rates as detailed next.

### Polarisation experiments

Polarisation experiments were performed using an automated computer-controlled variable resistor as previously described [6]. Single (forward) polarisation sweeps (See section: Hydrogel in cathode chamber) were carried out by applying 60 resistance values in the range of 1 MΩ down to 3 Ω, and each resistance was connected for 5 min.

For the bi-directional sweeps (discussed in section: Effect of anolyte flow rate on bi-directional polarisation), 60 resistance values were applied from 1 MΩ down to 3 Ω with a sample rate of 5 min for each value before the 60 resistance values were swept back up from 3 Ω to 1 MΩ. There was no ‘rest period’ between sweeps and so effectively the MFCs were held at 3 Ω for 10 min (5 min forward, 5 min reverse). Each bi-directional test lasted 10 h from start to finish.

### Data collection and calculations

MFC output was recorded in volts (V) against time by using an ADC-22 Channel Data Logger (Pico Technology Ltd., Cambridgeshire, UK). Recorded data were processed and analysed using the GraphPad Prism version 6 software package (GraphPad, San Diego, California, USA).

The current (I) in amperes (A) was calculated using Ohm's law, I = V/R, where V is the measured voltage in volts (V) and R is the known value of the external load resistor in ohms (Ω). Power (P) in watts (W) was calculated by multiplying voltage with current; P = IV. Power density was calculated in terms of anode volume; P_Density_ = P/α, where α is the anode chamber volume in metres^3^ (m^3^).

**Fig. 2:**
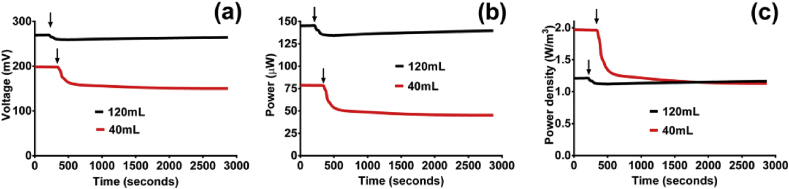
The effect of a 20 mL injection of air into different size MFCs; (a) voltage, (b) power and (c) power density (data is mean, n = 3).

**Fig. 3:**
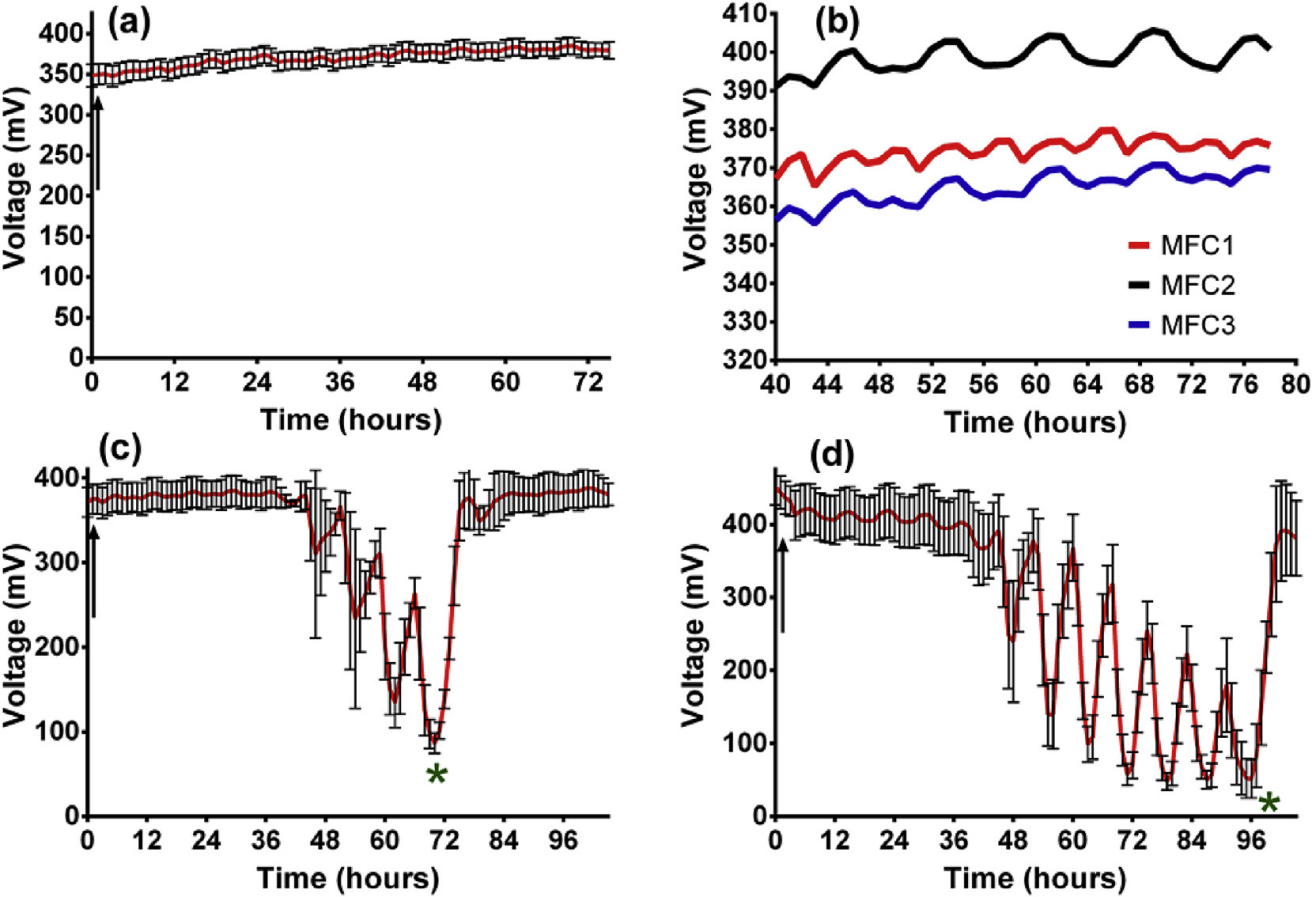
MFC voltage (under 500 Ω external resistance) in response to 4-h flow of air at different airflow rates through anode chambers; (a) air pumped at 126 mL per hour, (b) 126 mL magnified showing the three MFCs of the triplicate, (c) air pumped at 252 mL per hour, (d) air pumped at 1.1L per hour. Arrows indicate when airflow rate was initiated. Asterisk (*) indicates when anode was flushed with fresh anolyte. [(a), (c) and (d) data = mean and SD (n = 3)].

All data presented are the mean of 3 triplicate MFCs (with the exception of Fig. 3b that shows the three individual MFCs). Where relevant, error bars have been included with the exception of Fig. 2 where they have been omitted to improve clarity.

### Liquid analysis

Conductivity was measured using a Jenway 470 conductivity meter, pH readings were made using a Hanna HI2211 pH meter. COD was determined using the potassium dichromate oxidation method (COD test vials, CamLab, UK) and analysed using Lovibond MD200 photometer. The COD of the feedstock used in all experiments was approximately 1720 mg O_2_/L.

### Introducing hydrogel powder in MFC chamber

Six large terracotta MFCs (Table 1) were operated for a month with artificial wastewater (See section: MFC construction and operation) at a flow rate of 5.2 mL per hour before polarisation sweeps were performed. Following polarisation the cathode chambers of three of the MFCs were filled with 8g of hydrogel powder (Stockosorb, Germany). The MFCs were operated for a further 2 months before polarisation sweeps were performed again.

## Results and discussion

### The effect of air on performance

It is inevitable in a continuous flow wastewater treatment environment that oxygen will be present in the liquid. For MFCs positioned in such an environment it is important to understand how they might respond to the presence of oxygen. A number of studies using conventional proton exchange membranes have looked at the effect of oxygen on MFC performance but this is the first time that ceramic cylindrical MFCs have been used to assess the effect, and under conditions that resemble operation at a sequencing batch reactor (SBR). During standard operating conditions, the porosity of the ceramic material will allow partial oxygen to penetrate towards the anode electrode [36]. This will result in a lower open circuit voltage (OCV) because oxygen will contribute to a more positive anode redox potential. However, in a cylindrical configuration when the circuit is closed and electrochemical reactions proceed, in theory less oxygen will infiltrate the anode chamber because it is consumed during the oxygen reduction at the cathode. Interestingly, this could mean that the OCV in many studies using ceramic MFCs is understated and not representative of what might be under closed circuit conditions. This is because, despite ambient conditions being the same, the environment is altered through closed circuit operation. The purpose of the current study however, was the effect of oxygen on closed circuit operation with sequential batch reactor (SBR) operation being the main motivation for the experimental design.

#### Air pumped into the reservoir bottle

The first test was to pump air at 4-h intervals into the feed bottle and monitor whether there was any response from the MFCs by the time the feedstock had reached the anode chambers. There was no noticeable effect on electrical output of any of the MFCs and they all performed stably over time (data not shown). This demonstrated that the journey from the reservoir bottle to the MFC was sufficient so that any dissolved oxygen either dissipated or was insufficient to induce an effect.

#### Single injection of air directly into anode chamber

In order to observe how the MFCs respond to a single one-off influx of air, using a syringe, 5 mL and then 20 mL of air was injected directly into the MFCs. The lower 5 mL influx of air resulted in negligible change to electrical output (data not shown) however when 20 mL was injected the larger MFCs dropped 8% in power [3% drop in voltage] (Fig. 2a and 2b). The smaller fuel cell displayed a more significant drop, with a decrease in power of 42% (19% voltage). Following the drop, the MFCs stabilised before gradually recovering. When the power was normalised to chamber volume the effect is more marked in the smaller MFC, which initially has superior power density, but drops to below the larger MFC (Fig. 2c). The greater effect on the smaller MFCs is linked to the proportionately smaller volume of liquid containing dissolved oxygen, making it both quicker to saturate and unsaturate, and a higher proportion of electrode surface exposed to the dissolved air. Furthermore, by proportion, a larger area of chamber was flushed with air which could also negatively impact on the electro-active planktonic organisms.

Consequently, if the function of the MFCs was to sense the environment as a biosensor, for example to alert to the presence of oxygen, smaller MFCs are preferable because they demonstrated higher sensitivity and an immediate response time. MFCs are a natural biosensor because their output mirrors the microbial responses to the environment. To date, there are a number of reports of MFCs in a sensing role e.g. to monitor biological oxygen demand (BOD) [46], nitrate [31], toxic components [47] and other parameters [17]. MFCs have been employed to detect oxygen but these have been using oxygenated liquids fed to the cathode chamber [41]. This type of configuration would not be suitable if the presence of oxygen in the anolyte was of interest. The current study has shown that the response of the anodic biofilm to air could potentially serve a sensing purpose without harming the system.

For MFCs that need to be robust against a fluctuating environment, perhaps in the field of wastewater treatment, these data suggest that larger volume MFCs are more desirable. A more thorough examination was carried out next by subjecting the MFCs to longer periods of airflow.

#### 4 h on/4 h off flow of air into anode chambers

In order to mimic the sequential aeration conditions inside a SBR, two pumps were alternated every 4 h. Firstly, anolyte was pumped through the anode chambers for 4 h before air was pumped through the anode chamber for another 4 h; this continued in 4-h cycles. Fig. 3 shows the behaviour of three MFCs during the cyclic pumping. For the periods where air was flowing into the chamber (at 126 mL/h) the voltage did drop, but only a few mV (<2%, Fig. 3a) and the MFCs were not negatively affected by the flow of air through the anode chamber. To highlight this, Fig. 3b shows a magnified area of the three individual MFCs and the smooth fluctuations during the air followed by feed cycles. The flow of air was then increased to 252 mL/h and initially there was no detrimental impact on MFC performance (Fig. 3c). However, after 43 h the MFCs began to decline and with each new influx of air, the decrease became more exaggerated. After approximately 72 h the anode was flushed with fresh anolyte (indicated by asterisk in Fig. 3c) and the MFCs recovered immediately. When the airflow rate was increased yet further to 1.1L/h, the same pattern was observed where there was some stability before the staggered drop with the first significant decline occurring after 45 h.

To test the robustness of the microorganisms, the cycle was allowed to continue for another 25 h before the MFCs were flushed with fresh anolyte and the airflow rate dropped back to 126 mL/h (as indicated by the asterisk in Fig. 3d). Again the MFCs recovered immediately demonstrating that the constant flow of air did not harm the microbial community responsible for producing power. The reduction in power occurred when the air was flowing and each time the feedstock was introduced the MFCs tried to recover. However, performance did not recover fully to previous levels, because the anolyte flow rate was insufficient to purge the anode chamber of depleted feedstock. Prior to this point the air/oxygen was not inhibiting and the MFCs behaved in a stable manner. However, the air was accelerating the breakdown of organic matter, which is reflected by the decline in electrical output as the feedstock becomes depleted.

With each new air introduction the drop becomes more severe because the anolyte has been further depleted, a factor accentuated by the aerobic/facultative organisms present. The COD was measured at two points during the erratic behaviour and COD had dropped by 91% (asterisk in Fig. 3c) and 96% (asterisk in Fig. 3d) by the lowest point. When the MFCs were flushed with fresh feedstock the voltage quickly returned to the stable operating output thus demonstrating that the exposure to air had no long-term ill effect on the biofilm.

During the 4-h periods when air was flowing, there was no supply of feedstock but the liquid present in the anode chamber during this time was initially nutritious enough to maintain output. However as depletion was accelerated, airflow indirectly became inhibitory to the microbes. When feedstock was reintroduced every 4 h at the flow rate of 5.2 mL/h the electrical output began to pick up but the drops were more severe with each 4-h cycle because effectively a higher proportion of anolyte in the chamber had become exhausted. Furthermore, it is likely that microbes may have started growing inside the feed tube resulting in reduced COD entering the MFC. However, when the anolyte flow rate was temporarily increased (1.1L per hour) in order to replenish the chambers (as indicated by the asterisk) the MFCs quickly recovered.

**Fig. 4:**
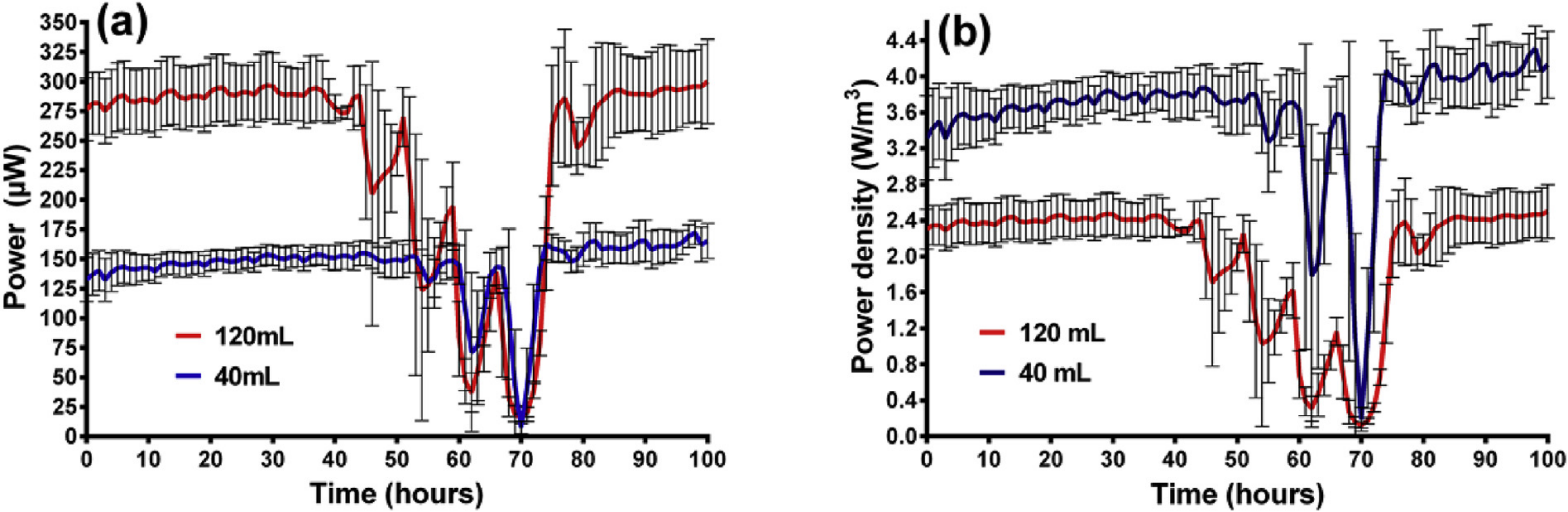
Comparison between large (120 mL) and small (40 mL) MFCs when subjected to 4 h periods of airflow through anode at 252 mL per hour: (a) power, (b) power density (data: mean and SD, n = 3).

The recovery can be seen in Fig. 4a, which shows a comparison in the power output of large and small MFCs in identical conditions where the airflow rate was 252 mL/h in 4-h intervals. Interestingly the smaller MFCs took longer (57 h compared to 43 h) for the decline to take effect. This is because the HRT was shorter for the small MFCs (8hrs) than for the large ones (23hrs) and so there was a better recycling retention of fresh feedstock. This is useful to know when considering MFCs for the role of receiving influent from a sequential batch reactor where air is pumped directly. This study demonstrates that the only effect that the cyclic provision of air has on the MFCs is to accelerate the breakdown of COD, which ultimately results in a decline in power. Focussing on the power illustrates how well the MFCs recover after what could be deemed a hostile environment for the bacteria. For example the power density (Fig. 4b) drops by 96% in the small and 94% in the large MFCs but recovers fully when fresh feedstock is flushed into the system (at approx. 70 h). The robustness of the organisms is highlighted by the fact that the MFCs have fully recovered within just 2 h.

Although no other gases were tested in the current study, there have been other reports to suggest that gas bubbles can improve performance; e.g. bubbles of CO_2_ can improve mass transfer through agitation [4]. Furthermore, the presence of oxygen can help MFCs access fuels that normally would not be treated in a strictly anaerobic environment [3].

### Effect of anolyte flow rate on bi-directional polarisation

When operating MFCs in continuous flow, the flow-rate is vital for maintaining biofilm stability and efficient delivery of nutrients [19]. Too high a flow rate i.e. a lower hydraulic retention time (HRT) will result in an inadequate breakdown of organic matter, and could result in shearing the biofilm, which implies higher maintenance requirements and slow biofilm maturation [29]. A longer HRT will ensure organic matter is more efficiently removed but too long could impact on the biofilm as the organisms receive depleted feedstock. A further complication to operation is the design of the MFC and how the system is configured. In the current study feedstock was fed into the bottom of the anode chamber with the treated effluent leaving via the outlet at the top (Fig. 1b). Therefore, organisms colonising the top half of the electrode will have been receiving a more depleted feedstock than those in the lower half of the anode. The nutrient composition particularly received by those at the top of the anode will very much depend on the flow rate. Another factor that flow rate brings is the amount of dissolved oxygen i.e. higher flow rates will deliver more oxygen than the low flow rates [14].

**Fig. 5:**
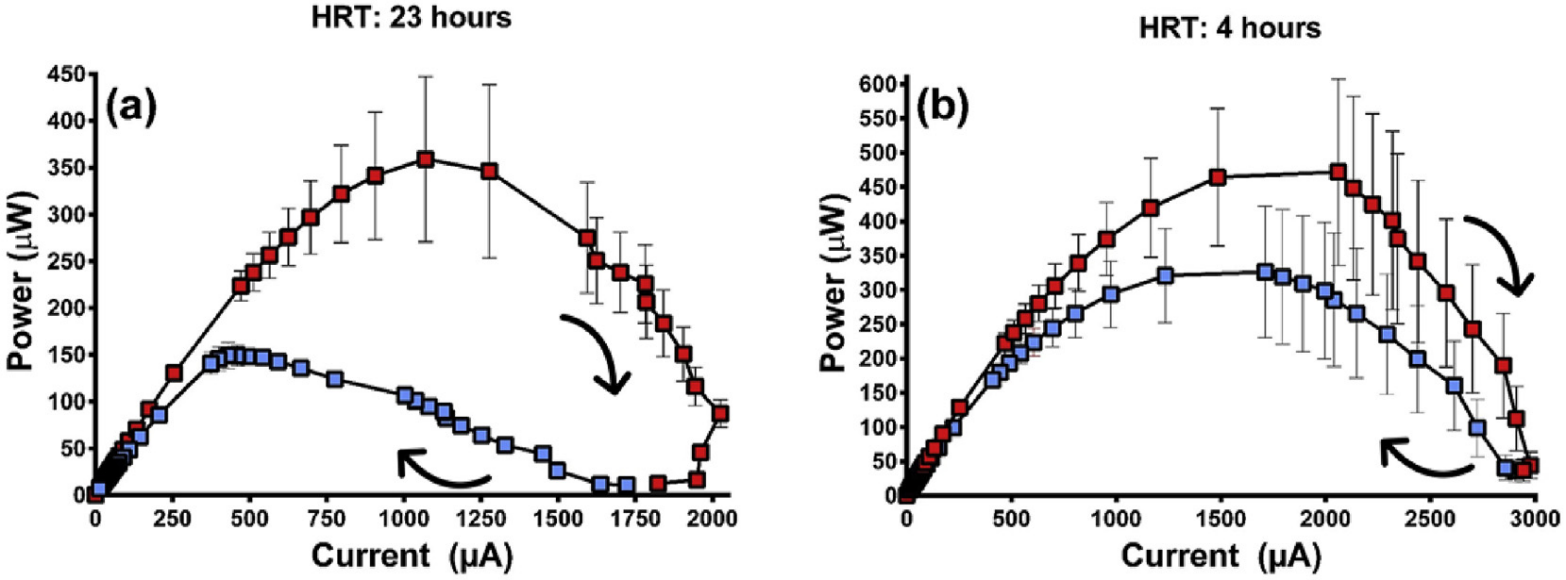
The effect of anolyte flow rate on bi-directional power curves generated by 120  mL MFCs at either; (a) 5.2 mL/h or (b) 30 mL/h (data: mean and SEM, n = 3).

The bi-directional polarisation sweep is a useful tool for assessing the health of the MFC [48], stability of materials [33] and is important with regards to selecting the right analytical methods [7]. For this study, the technique was used to examine the effect of flow rate on the stability of biofilm over time. At the lower flow rate, which equates to 23 h HRT (Fig. 5a), the point of maximum power (MPT) drops by 58% demonstrating that the environment has altered considerably over the course of the first resistance sweep, to the detriment of the microbial community. Over the faster flow rate (HRT 4 h) the MPT still drops between the first sweep and the second but the decline is less severe at 31% (Fig. 5b). This demonstrates that at a higher flow rate with more efficient replenishment of nutrient the microorganisms respond positively as epitomised by healthier power curves. In the case of these MFCs, it is hypothesised that higher flow rates may have generated healthier curves with less hysteresis. The bi-directional polarisation sweep could then be a useful tool for determining optimal flow rates based on the extent of hysteresis and this is an area for future work.

### Hydrogel in the cathode chamber

One of the logistical problems associated with ceramic cylindrical MFCs that incorporate internal cathodes is how to ensure efficient contact between the cathode and the internal wall. Should the cathode peel away or come apart, proton transfer is inhibited and power output diminishes. A number of methods have been adopted to try negating the issue and generally involve holding the cathode material against the wall with a material such as acrylic rings [24]. These methods can cause tearing of the cathode and may inhibit the availability of oxygen at the electrode surface. A new technique was trialled in this study using hydrogel powder. While this is not the first time hydrogel has been used in MFCs, it is the first time it has been employed to improve contact and as a mechanism for collecting catholyte.

**Fig. 6:**
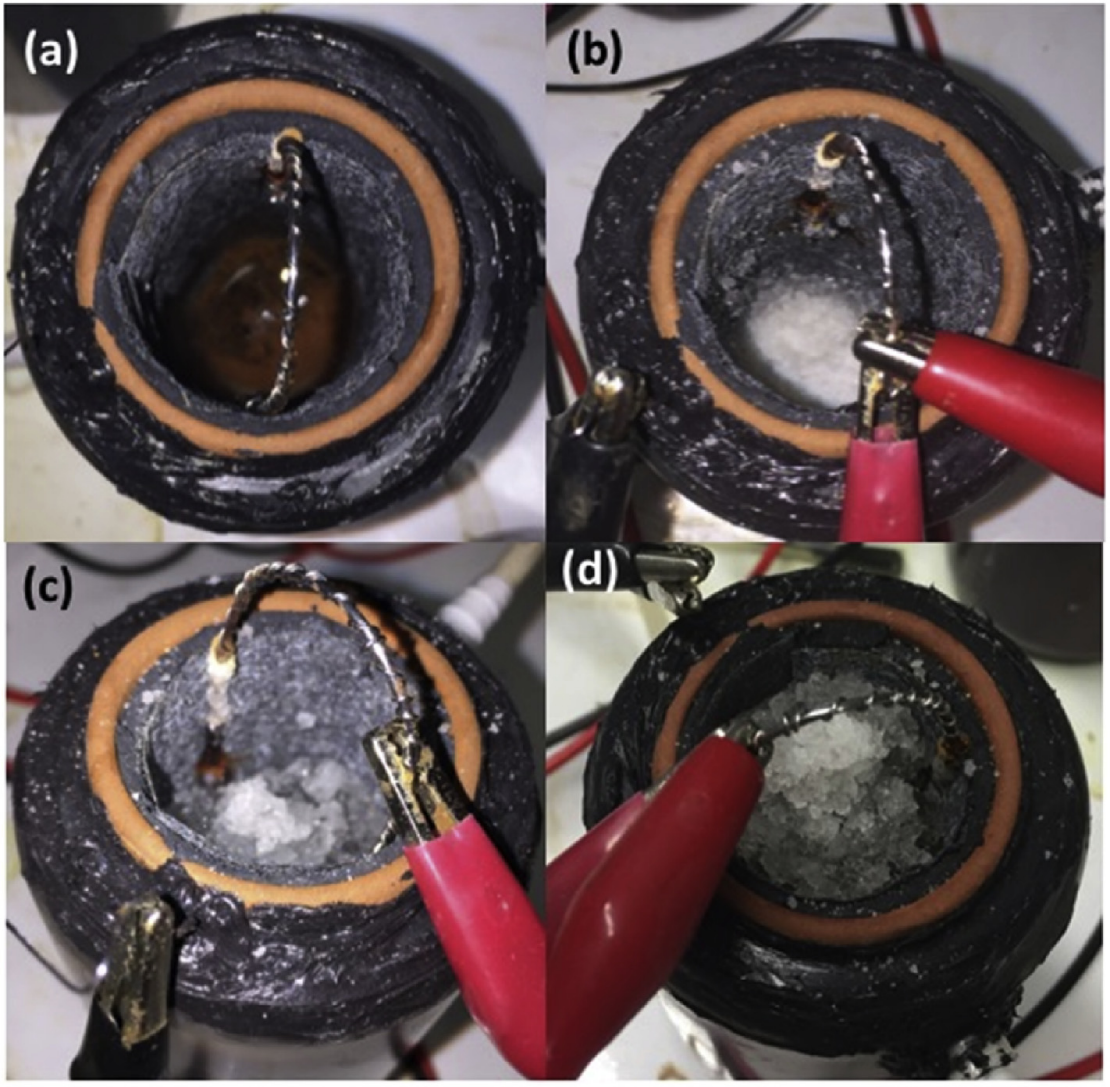
The cathode chamber of MFCs; (a) before hydrogel powder added, (b) Hydrogel powder added (day 1), (c) after 1 month, and (d) after 2 months.

Hydrogel in its powdered form is used in the cultivation of plants, it retains water in the earth and can hold 150 times its own weight making water more accessible to the roots of plants. Forms of hydrogel have been trialled in MFCs for other roles such as a passive feeding mechanism [37] and as a bridge between electrode and ion exchange membrane to increase cathode potential [49]. In the current study hydrogel powder was poured into the inner cathode chamber after draining the catholyte (Fig. 6a).

**Fig. 7:**
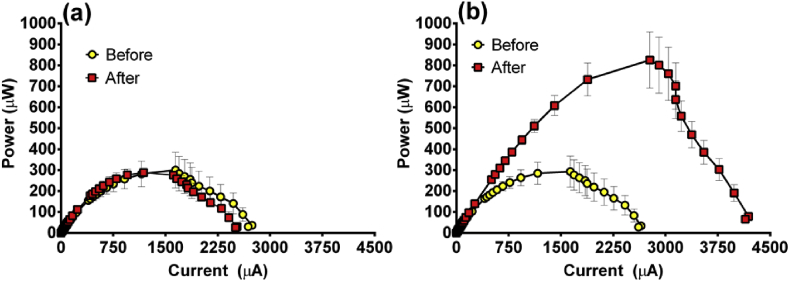
Power curves showing the effect of adding hydrogel powder into the cathode chamber (a) control MFCs that did not have hydrogel added, before and 2 months later and (b) MFCs that did have hydrogel added, before hydrogel addition and 2 months later (data = mean and SEM, n = 3).

Prior to the addition of hydrogel, a polarisation sweep was performed on 6 MFCs; 3 which would have hydrogel powder added and three controls that wouldn't. The power curves of all 6 MFCs prior to the addition of hydrogel were comparable (yellow symbols, Fig. 7a and Fig. 7b) where the average of the hydrogel MFCs was 293 μW (1634 μA) before the addition of hydrogel compared to 300 μW (1642 μA) generated by the MFCs that would remain without hydrogel. Following the polarisation experiment the hydrogel was added to the three hydrogel MFCs and for the next 2 months all MFCs were maintained in identical conditions. During this time the hydrogel became noticeably more swollen as it absorbed any catholyte that was being produced (Fig. 6) and after 2 months had swollen to the brim of the chamber (Fig. 6d). Interestingly the conductivity of the hydrogel was higher (15 ± 1 mS/cm) than that of the liquid catholyte (8 ± 1 mS/cm) and the pH was also higher (10.5 ± 0.2) than the liquid catholyte (9.1 ± 0.2).

After 2 months, polarisation experiments were performed again on the six MFCs and those without hydrogel addition remained comparable to the output recorded 2 months earlier (Fig. 7a). Those MFCs that had the addition of hydrogel displayed significant improvement (Fig. 7b) with the maximum power transfer point more than two and a half times higher (825 μW [2775 μA]). This demonstrates that hydrogel can be used to aid performance in future research using ceramic cylinder MFCs by improving the contact between ceramic and cathode. Hydrogel serves the purpose when used in agriculture of reducing the effects of evaporation and leaching by retaining the moisture. In the current study it retained and helped the accumulation of catholyte resulting in a more concentrated sample as reflected by the increased conductivity and pH. Further work will investigate whether the catholyte-hydrogel material might serve a useful purpose such as in an antimicrobial capacity [9] or in the role of atmospheric carbon capture [10]. In addition, further work will investigate the longevity of the hydrogel system and whether it continues to aid performance or whether it will need replacing.

## Conclusions

In this study, ceramic cylindrical MFCs were used to investigate three operational challenges (i) the effect of airflow through the anode, (iii) hydraulic retention time and its effect on biofilm stability and (iii) improvement of the cathode (using hydrogel powder)

i. Generally, when considering MFC operation, the user will go to lengths to limit the penetration of air into the anode chamber. This is because oxygen consumes electrons causing a decline in electrical output and it can harm strict anaerobes. We have demonstrated that the influx of air had a negligible effect on biofilm health and power output. When air was bubbling through the anode chamber the power dropped by just 1% and recovered quickly when the airflow stopped. However, the presence of air did accelerate the breakdown of organic matter and eventually at around 44 h the MFCs declined through nutrient depletion. When replenished the MFCs responded instantly demonstrating that there was no permanent damage to the biofilm.
ii. Bi-directional polarisation curves highlighted the importance of hydraulic retention time on biofilm stability. The HRT of 23 h proved too long as demonstrated by a 58% drop in maximum power between first and the second polarisation sweeps. When the HRT was 4 h the MFCs performed much better on the return sweep with just a 31% drop in maximum power. We propose that the bi-directional polarisation method can be used to determine optimal operational conditions such as flow-rate.
iii. When hydrogel powder was incorporated into the cathode chamber there was a 182% increase in power output after 2 months compared to zero improvement for MFCs without hydrogel. As the material swelled with the electrochemically produced catholyte, a better contact between cathode and ceramic surface was maintained alongside the production of a potentially beneficial alkaline gelatinous material.
